# Adjusting the british triage system for dental care in South Korean correctional institutions: a cross-sectional study

**DOI:** 10.1186/s12903-023-03207-4

**Published:** 2023-07-24

**Authors:** Ilkwang Hwang, YoungHa Song, Hee-Kyung Park

**Affiliations:** 1 Department of Oral Medicine and Diagnosis, School of Dentistry and Dental Research Institute, Seoul National University, #101, Daehak-ro, Jongro-Gu, Seoul, 03080 Korea; 2 Department of Preventive and Social Dentistry, School of Dentistry and Dental Research Institute, Seoul National University, Seoul, Korea

**Keywords:** Triage, Emergency, Dental Services, Oral health, Correctional institutions, Public Health Dentistry

## Abstract

**Background:**

The oral health status of inmates in South Korean correctional institutions is poor, mainly due to limited resources and an unestablished triage system. Hence, this study aimed to develop a newly structured dental triage system for South Korean correctional institutions, using the British triage system as a reference.

**Methods:**

This study included 32 public health dentists working at correctional institutions in South Korea in 2020, accounting for the entire population of public health dentists that year. Data on the dentists’ evaluation of resources and perceptions of dental service items were collected using a self-administered online survey including 19 dental service items from the British triage system to assess the level of agreement on dental triage items. All responses were recorded within 1 week of request, and a hierarchical cluster analysis was performed to develop a new dental triage system.

**Results:**

The survey included 31 respondents working at 47 correctional institutions; 16, 14, and one respondent provided dental services at one, two, and three institutions, respectively. Among the correctional institutions, 2%, 74%, and 23% were the National Forensic Hospital, prisons, and detention centres, respectively. The hierarchical cluster analysis identified four adjusted dental triage categories: emergency, urgent, routine, and checkups, mainly in accordance with those in the British system, but a few items were reallocated. The new dental triage system was compared to the existing system and found to have higher specificity and sensitivity, indicating that it may be more effective at meeting the oral health needs of inmates in South Korean correctional institutions.

**Conclusions:**

This study developed a newly structured dental triage system by adjusting the British system and evaluated its efficacy compared to the existing system. The new system may help improve the oral health status of inmates in South Korean correctional institutions by providing a more organized approach to dental care provision.

## Background

Access to dental services for the general public has improved owing to the increasing number of dental personnel and the introduction of dental insurance systems [1–5]. However, there are still vulnerable groups with special needs for oral health [6]. Generally, inmates in correctional institutions tend to have worse oral health compared to non-incarcerated individuals, often requiring more intensive care and treatment [7–13]. Epidemiological data have shown differences between the nature of oral conditions and self-rated oral health for incarcerated individuals, further justifying the need for a triage system that differs from other services [13].

South Korea is no exception in that the oral health status of inmates is poor compared with that of non-incarcerated individuals [13]. Overall, 53 local correctional institutions in South Korea, each with an affiliated clinic or medical department, provide healthcare services to inmates. Additionally, the Ministry of Justice operates a single National Forensic Hospital (NFH) mainly for psychiatric evaluation and forensic treatment. Inmates receive dental care in these affiliated clinics equipped with scarce human, financial, and dental resources. Most dentists in these clinics are male public health dentists (PHDs) undergoing alternative mandatory military service [14]. Furthermore, according to South Korean criminal law and ordinance, incarcerated persons are disqualified from receiving the National Health Insurance Service (NHIS) after incarceration, and the Ministry of Justice covers their medical expenses. Thus, although dental services are provided free of charge to inmates in correctional institutions, the quality of care seems questionable.

Dental triage systems can provide appropriate dental services with limited resources within correctional institutions. A dental triage system was first implemented at the Hydebank Wood Prison and Young Offenders Centre in the United Kingdom (UK) [15]. Triage in this institution was operated using a three-strand system: induction triage, screening examination, and prison landing referrals based on several studies that have introduced triage categories and corresponding timeframes in correctional institutions [16, 17]. These studies have suggested four triage categories according to the importance of the timeframe: emergency, urgent, routine, and checkup. An evaluation of the Hydebank Wood Prison triage selection during subsequent treatment resulted in 95% of patients being assigned to the correct triage category and 72% receiving adequate treatments according to the timeframe standard in the UK [15]. Additionally, another detention institution reported that the implementation of the above triage system reduced the waiting time for dental services [18].

In contrast to the British system, the oral health of South Korean inmates has been relatively neglected due to insufficient resources and an unestablished triage system. There is an urgent need to develop a South Korean dental triage system with prepared protocol standards. Therefore, this study explored the possibility of developing a new triage system in South Korea through dentists’ evaluation of resources and perceptions of dental service items using the British triage system as a reference.

## Methods

### Study design, setting, and participants

This cross-sectional study was conducted via an online survey administered in March 2020. The study population comprised 32 PHDs who provided dental treatment at correctional institutions nationwide in South Korea, including the first author. These PHDs represented the total number of PHDs working in South Korean correctional institutions during the study period.

The eligibility criteria for PHDs included: (i) individuals with dental expertise, possessing valid dental licenses in South Korea; (ii) those without restrictive conditions after government-conducted physical examinations and were required to undergo compulsory or equivalent military service; (iii) individuals with ≥ 6 months of experience providing dental treatment in correctional facilities, ensuring clinical proficiency. In South Korea, PHDs are selected annually and serve up to 3 years with the option to transfer to other facilities yearly. Participants in this study were required to have started their service before April 2019 and had ≥ 11 months of service experience during the study period. PHDs who did not work in correctional institutions during the study period were excluded from this study.

**Fig. 1:**
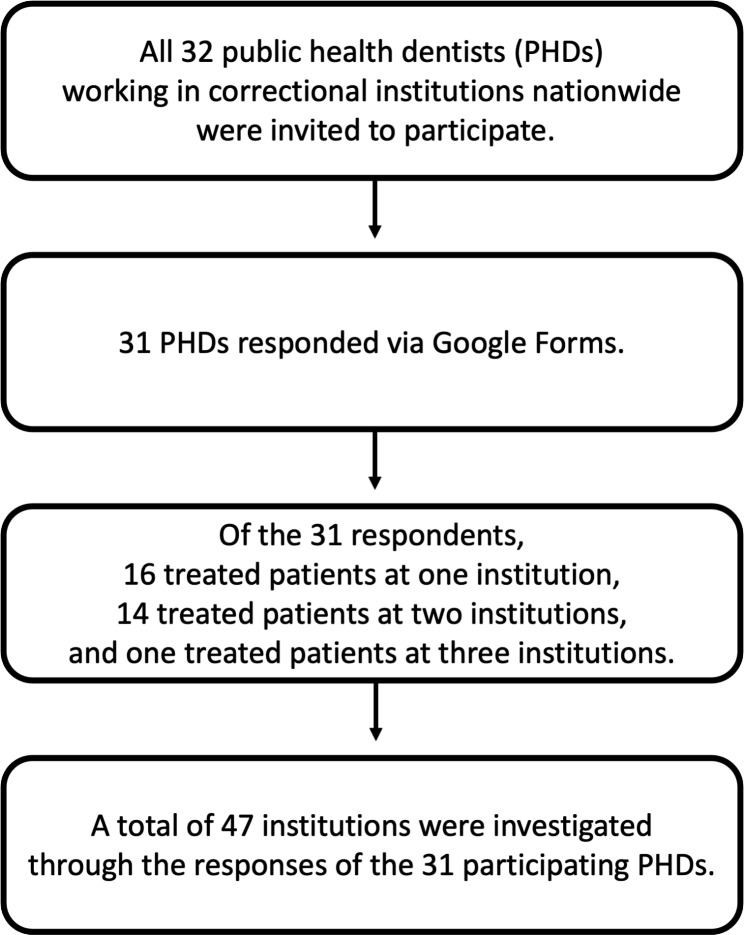
Flowchart of the study design and participants

The PHDs were invited to participate in the study using a link created through Google Forms. Details of the study population and participants are presented in a flowchart (Fig. 1). All participants provided informed consent prior to participation. This study was approved by the Institutional Review Board of the School of Dentistry, Seoul National University, Seoul, Republic of Korea (IRB No. S-D20200013).

### Data collection

The survey was designed to evaluate dental resources available in each correctional institution and determine the perception of PHDs working with them. The questionnaire included 19 dental service items from the British triage system, and each item was labelled using the original category name of the British system for the convenience of the analysis and description (Table 1). For example, since ‘bleeding post-extraction’ belongs to the ‘emergency’ British triage category, it was coded as E1. The questionnaire had three questions about each dental service item. The first question asked about the sufficiency of resources available to treat each item, and responses ranged from ‘very sufficient’ to ‘not sufficient at all’ in four levels. The second question asked what categories of dental service items were currently being provided at each institution. The third question inquired about the perception of PHDs regarding the proper classification of each item. There were four alternative answers for the latter two questions: (i) as soon as possible (within 4 h of reporting), (ii) within 1 week of reporting, (iii) in a normal session (within 1–2 months of reporting), and (iv) no treatment right away and a checkup every 6 months. Terms such as emergency, urgent, and routine were not used explicitly to prevent social desirability bias due to the literal meaning of the category itself. The survey was self-administered and conducted online, and all responses were recorded within 1 week of request. The survey did not involve face-to-face interactions or interviewers, and no survey pre-test was conducted, considering the small number of participants.

**Table 1:** Distribution of responses on dental triage items

Item	Description of item	Distribution of responses on dental triage items (%)											
		Resources for treatment (n = 47)^a^				Status of the treatment provided (n = 47)^b^				PHDs’ perception of how to treat (n = 31)^c^			
		VS	S	NS	NSA	Em	U	R	C	Em	U	R	C
E1	Bleeding post extraction	4	32	**55**	9	38	**40**	21	0	**83**	17	0	0
E2	Oro-facial swelling – increase in size, periorbital, and swallowing	0	19	**70**	11	28	**47**	26	0	**63**	33	3	0
E3	Trauma – lacerations, bony fractures	0	4	**68**	28	**40**	34	26	0	**87**	13	0	0
E4	Severe trismus	2	2	**64**	32	23	**47**	30	0	**53**	43	3	0
U1	Dental abscess	0	28	**55**	17	19	38	**43**	0	37	**60**	3	0
U2	Irreversible pulpitis - not controlled by analgesics	0	23	**55**	21	11	40	**47**	2	43	**53**	3	0
U3	Fractured tooth – dentine or pulp involved	0	28	**55**	17	19	**40**	**40**	0	**53**	43	3	0
U4	Pericoronitis	2	43	**47**	9	4	40	**49**	6	30	**43**	23	3
U5	Oral medicine – long-standing ulcer or problem	2	4	**74**	19	4	40	**49**	6	20	**47**	23	10
U6	Acute necrotizing ulcerative gingivitis	4	13	**70**	13	9	35	**57**	0	30	**60**	10	0
U7	Fractured denture	0	4	43	**53**	0	23	**62**	15	13	**50**	23	13
R1	Dental decay	4	43	**45**	9	0	40	**49**	11	10	**43**	37	10
R2	Reversible pulpitis - controlled by analgesics	2	**47**	36	15	4	40	**49**	6	17	**47**	33	3
R3	Root canal treatment to be completed	0	23	**51**	26	2	24	**54**	20	10	33	**43**	13
R4	Gingivitis	4	**51**	38	6	4	36	**47**	13	3	**33**	**33**	30
R5	Request for prosthetics, cosmetic to orthodontic treatment	0	4	28	**68**	0	9	36	**55**	3	10	33	**53**
C1	Patient is a current attendee with a personal GDP and no outstanding treatment	30	**33**	26	11	2	15	30	**52**	3	10	10	**77**
C2	Patient is happy with their teeth	33	**35**	24	9	2	13	30	**54**	3	3	13	**80**
C3	Patient is not interested in receiving treatment	33	**37**	22	9	2	13	26	**59**	3	3	17	**77**

PHD, Public Health Dentist; GDP, General Dental Practitioner

VS: Very sufficient; S: sufficient; NS: Not sufficient; NSA: Not sufficient at all

Em, Within 4 h of reporting; U, Within 1 week of reporting; R, Within 1–2 months of reporting, in a normal session; C, No treatment right away – checkup every 6 months

^a^One missing value for C1, C2, and C3

^b^One missing value for U6, R3, C1, C2, and C3

^c^One missing value for all items (One participant did not respond)

Bold font represents the most frequent response in the category

### Statistical analysis

Descriptive analyses were performed using frequency tables. Agglomerative hierarchical clustering was then performed using Ward’s method to minimize the variance between members within the cluster [19]. Finally, the structural hierarchy of the cluster analysis was drawn on a dendrogram in a tree format for a better visual understanding of the categories. Statistical analyses were performed using IBM Statistical Package for Social Sciences Statistics ver. 25.0 (IBM Corporation, Armonk, NY, USA).

## Results

### Participant characteristics

Of the 32 eligible PHDs, 31 completed the survey. The survey responses were analysed, and it was found that 16 respondents provided dental services at one institution, 14 respondents at two institutions, and one respondent provided services at three institutions. Therefore, a total of 47 correctional institutions were investigated via the 31 PHDs who responded to the survey (Fig. 1).

All the respondents were male PHDs < 35 years old, and 26 (84%) were general dental practitioners. The remaining five respondents were specialists, including one in periodontology, one in conservative dentistry, two in orthodontics, and one in pediatric dentistry.

### Capacity and types of correctional institutions

The capacity of each correctional institution was as follows: 0-499 (10; 21%), 500–999 (12; 26%), 1,000–1,499 (13; 28%), 1,500-1,999 (8; 17%), 2,000–2,499 (2; 4%), and 2,500-2,999 (2; 4%). Among the correctional institutions, 2%, 74%, and 23% were NFH, prisons, and detention centres, respectively.

### Resources and treatment status for each triage category item and PHDs’ perceptions

The responses showed whether each institution had sufficient resources available to attend to each dental item (n = 47). A lack of resources was observed for most items that required treatment. While responding to emergencies is crucial, the survey results revealed that all four emergency items (E1, E2, E3, and E4) were marked as ‘Not sufficient’ or ‘Not sufficient at all’ by a majority of respondents (E1: 64%, E2: 81%, E3: 96%, E4: 96%). This indicates that the institutions lacked adequate resources to provide treatment for these emergency cases. The proportion of participants who responded that the available resources were “not sufficient at all” was high for some items, especially for items U7 (53%) and R5 (68%). Contrastingly, very few to no participants responded that the resources for treatments were “very sufficient” (Table 1).

Each institution (n = 47) provided dental services for each item, and 40% or less of each of the four items corresponding to an emergency were treated as emergencies (E1, 38%; E2, 28%; E3, 40%; and E4, 23%). Conversely, some institutions treated items of the checkup category as emergencies (C1–C3, 2%). Although appropriate treatment is required for each item, some institutions did not properly attend to emergency or urgent items and only dealt with checkups (Table 1).

Thirty PHDs responded to the question on how to treat each triage item. Among the four emergency items, 83% and 87% of the respondents thought E1 and E3 required immediate attention within 4 h of reporting. However, only 53% of respondents thought that E4 required immediate attention. Items that did not reach the maximum number of answers for each category were also noted. For example, responses for U3 in the urgent category and items R1, R2, and R5 in the routine category did not match the corresponding timeframe. Responses that U3, R1, and R2 should be treated sooner and R5 a little later were not placed in the correct categories. The answers to these questions are summarized in Table 1.

### Cluster structure

**Fig. 2:**
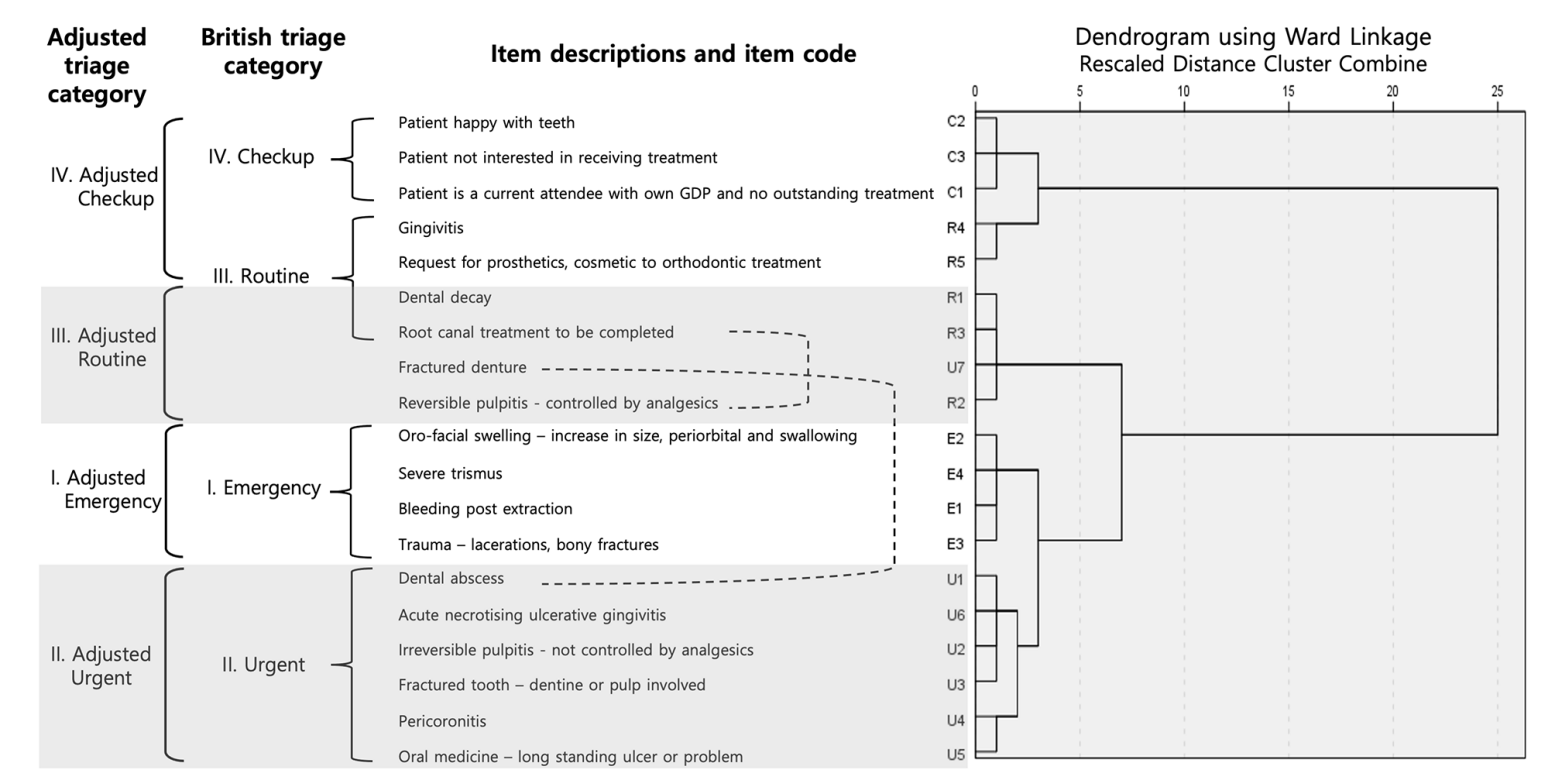
Dendrogram from the cluster analysis of triage category items GDP, General Dental Practitioner

From the dendrogram’s splitting pattern, the first division was made by clustering C1–C3 and R4–R5 into a single branch, leaving other items in the main trunk (Fig. 2). Then the next branch, comprising R1–R3 and U7, was separated from the stem. Lastly, a cluster was formed with the division of E1–E4 and U1–U6 into homogenous category items. The classification of items into categories was generally comparable to that of the British Triage System, with minor exceptions. For example, the ‘checkup’ cluster was grouped with two routine items on the first split of the dendrogram. Furthermore, one urgent item was assigned far away from its peer group with the ‘routine’ cluster. Lastly, the items were grouped into brackets with four domains named the new triage category, in reference to the existing British triage category divided into four domains.

## Discussion

The explicit shortage of resources for dental services in South Korean correctional institutions highlights the need for this study. No full-time managerial-level manpower is employed for dental services, and all dentists currently working in South Korean correctional institutions are time-bound and underpaid PHDs [14]. Furthermore, this PHD dispatch system is unique to South Korea, as it is a result of the ceasefire against North Korea since the Korean War. According to South Korea’s Military Service Act, all non-disabled and able-minded adult males must engage in compulsory or equivalent military service. Hence, those with medical or dental licenses may serve as public health doctors or PHDs for ≥ 3 years after completing basic military training instead of general military service. They are deployed to offer healthcare services in some national institutions, such as prisons, where it is difficult to recruit medical officers. However, the PHDs are employed only for short-term services with suboptimal authority, and their clinical experience may be insufficient. In this study, only 16% of the PHDs majored in a speciality during their training program, and 60% of these specialists majored in orthodontics and pediatric dentistry. The question remains whether their specialities are advantageous for dental treatment in correctional institutions. Moreover, half of the PHDs are working at more than one institution, which can be burdensome with commuting distance. Further, 53% of these institutions can accommodate more than 1,000 people, and 9% have a capacity of more than 2,000 people. This highlights the significant shortage of dental professionals in South Korean correctional institutions, further underscoring the need for improvements in the provision of dental services.

The resources for treating each item classified in the triage categories are reportedly insufficient. Among the items requiring appropriate treatment (not belonging to the checkup category), only one item (R4, gingivitis) had more sufficient treatment resources than insufficient resources. In particular, < 10% of respondents answered that there were sufficient resources for treating E3, E4, U5, U7, and R5, which may lead to poor quality of care in practice. Perhaps because of these scarce resources, the method of dealing with items in each institution deviated greatly from the British prison triage categories. Less than half of the respondents answered that dental treatment was performed according to the emergency timeframe for the four items classified as emergencies. Conversely, emergency treatment was performed for non-emergency items, especially urgent items, in some institutions. This raises questions about possible waste and inappropriate allocation of resources, considering the insufficient amounts of resources available. One possible reason is that the dental budget is a part of the overall medical department budget, which is also insufficient.

The PHDs’ perceptions of how to treat each item showed somewhat different results from the nominal categories. Compared with the treatment status currently conducted by each institution, the percentage of responses for the properly normative timeframe was higher. However, since South Korean correctional dental service environments lack resources and a triage system, the PHDs’ perceptions of the prescriptive timeframe did not match that of the British system. The results of the clustering analysis also supported this pattern. Some items in each category tended to be grouped into categories with a lower timeframe, as follows: (1) U7 was grouped with routine items (R1–R3), and (2) R4 and R5 were grouped with checkup items (C1–C3). However, PHDs may have made punitive judgments that people who did ‘bad things’ and were incarcerated do not deserve the due dental care and thus may have delayed the treatment timeframe compared to that of non-incarcerated citizens.

Although most respondents considered U7 urgent, 36% recognized it as a routine or checkup item. Perhaps because of this, it was grouped as more similar to a routine item. This may also be related to the fact that the respondents’ opinion that resources were ‘not sufficient at all’ was most frequent for U7 (53%) compared with the opinion that resources were ‘not sufficient’, which was the most frequent category for other urgent items. Additionally, U7 could have been associated with R5 (request for prosthetics and orthodontic cosmetic treatment) if the existing dentures could not simply be repaired as it required the manufacture of new prosthetics. R4 was grouped with C1–C3 in the clustering structure, and the opinion that R4 should be a checkup item was shared among 30% of the respondents. This may be because PHDs agree that R4 can be treated by alternative methods, such as tooth brushing and medication, instead of special interventions using scarce resources. In addition, its high prevalence among the general population may have given it a low triage ranking as one of the most common chronic diseases. R5 was also grouped as a checkup item that did not require treatment, and 53% had the opinion that it should be a checkup item. The South Korean NHIS covers ‘essential’ dental treatments for the general public; hence items not covered by insurance, such as dental prosthetics and orthodontic cosmetic treatments, can be considered ‘non-essential’. Therefore, the respondents may have been influenced by the NHIS coverage for triage categorization.

The National Health Service (NHS), similar to Korea’s NHIS, has long been operating in the UK; however, both services differ from one another. Patients must visit an NHS-designated registered dentist to get NHS coverage in the UK, while South Korean patients can get NHIS coverage from any dentist in the country. These may have caused a collective difference in the classification of the triage categories. However, the category changes observed with U7, R4, and R5 did not affect items in the emergency category, and only U7 was affected in the urgent category. Furthermore, these results did not conflict with the emergency and urgency standards specified by the British dental service for prisons [20–22]. Considering these, newly adjusted dental triage categories are required for South Korean correctional services. Dental care items can be classified into four categories: (1) adjusted emergency, (2) adjusted urgent, (3) adjusted routine, and (4) adjusted checkup. The items belonging to each category are shown in Fig. 2.

The first dental triage system for correctional institutions was trialled at Hydebank Wood Prison [15]. Based on this, the triage system was introduced in the National Association of Prison Dentistry UK handbook [23], and the British Dental Association suggests that prison dentists must be fully aware of its contents [20–22]. The UK has a three-strand triage system, which South Korea does not have. South Korean inmates are treated sequentially based on their order of application, and emergencies are only prioritized when reported through prison officers or by referral from a medical doctor. Whereas prisons under the Federal Bureau of Prisons in the United States have a triage system in which dentists determine immediate needs, routine, and accessory care [24].

This study had some limitations. First, the number of participants (n = 31) might have been insufficient for cluster analysis; thus, the study’s findings should be interpreted cautiously. Second, the study focused mainly on PHDs’ perception of the triage system and did not assess inmates’ dental needs, which is discordant with patient-centred care. Despite these limitations, the study also had several strengths. First, the findings are grounded in the study population data, as 97% of the PHDs who worked in South Korean correctional institutions during the study period responded to this survey. Second, these PHDs can be considered the only dental care experts in South Korean correctional institutions due to the lack of full-time regular dental officers. As of March 2020, 32 PHDs were deployed in correctional institutions nationwide, which was a significant increase from 9 to 17 PHDs in 2015–2017 and 2018–2019, respectively. Furthermore, this was the highest number recorded in recent years. Moreover, the 47 institutions investigated in this study included most of the 53 local correctional institutions and the sole NFH nationwide. These points provide ample strengths to the study, while also considering that this is the only survey on correctional dental services in South Korea.

Besides South Korea and the UK, developing dental triage systems for correctional institutions is also recommended in other countries, considering the inmates’ poor oral health. The investigation of this triage system in different contexts will lead to diverse research topics. However, the availability of dental services, which are subject to legal systems and availability of resources, is significantly different in each country. Thus, the triage system should be properly considered based on pre-existing findings. Additionally, despite the recent increase in the number of PHDs, private dental appointments paid for by the inmates, are still the main treatment pathway in each institution. Hence, whether the same triage system should be used among inmates who are willing to pay instead of receiving free in-house treatment is questionable. This also raises the ethical issue of whether only people with a financial allowance should afford dental care. Further research on the socioeconomic determinants of oral health, even in a disadvantaged society, is required.

Recently, there have been discussions about essential dental care due to the COVID-19 pandemic [25–27]. Expert discussions should be actively conducted to determine the essential treatment areas for which consensus is still lacking [25]. Furthermore, it is necessary to understand treatment barriers to improve dental access for underserved people [28].

## Conclusion

This study proposes new triage categories that consider the unique circumstances and limited resources in South Korean correctional institutions. These new triage categories can improve the provision of timely dental services and promote oral health among inmates. Further discussion and attention to this topic may lead to practical improvements in dental care for South Korean inmates.

## Data Availability

The datasets used and/or analysed during the current study are available from the corresponding author, Hee-Kyung Park, on reasonable request. The data are not publicly available due to the nature of this research and per the restrictions of our IRB.

## Abbreviations

NFH: National Forensic Hospital
NHIS: National Health Insurance Service
NHS: National Health Service
PHD: Public health dentists
UK: United Kingdom
